# Recent Progress on Covalent Organic Frameworks Supporting Metal Nanoparticles as Promising Materials for Nitrophenol Reduction

**DOI:** 10.3390/nano14171458

**Published:** 2024-09-07

**Authors:** Mohammad Dinari, Zaynab Golshadi, Parvin Asadi, Amie E. Norton, Katelyn R. Reid, Benson Karimi

**Affiliations:** 1 Department of Chemistry, Isfahan University of Technology, Isfahan 84156-83111, Iran; 2 Department of Medicinal Chemistry, School of Pharmacy and Pharmaceutical Sciences, Isfahan University of Medical Sciences, Isfahan 81746-73461, Iran; 3 Department of Entomology, Kansas State University, 123 W Waters Hall, 1603 Old Claflin Place, Manhattan, KS 66503, USA; amien@ksu.edu; 4 Department of Physical and Environmental Sciences, Texas A&M University Corpus Christi, Corpus Christi, TX 78412, USA

**Keywords:** covalent organic frameworks, heterogeneous catalyst, noble metal nanoparticles, nitrophenol reduction, aminophenol

## Abstract

With the utilization of nitrophenols in manufacturing various materials and the expansion of industry, nitrophenols have emerged as water pollutants that pose significant risks to both humans and the environment. Therefore, it is imperative to convert nitrophenols into aminophenols, which are less toxic. This conversion process is achieved through the use of noble metal nanoparticles, such as gold, silver, copper, and palladium. The primary challenge with noble metal nanoparticles lies in their accumulation and deactivation, leading to a decrease in catalyst activity. Covalent organic frameworks (COFs) are materials characterized by a crystalline structure, good stability, and high porosity with active sites. These properties make them ideal substrates for noble metal nanoparticles, enhancing catalytic activity. This overview explores various articles that focus on the synthesis of catalysts containing noble metal nanoparticles attached to COFs as substrates to reduce nitrophenols to aminophenols.

## 1. Introduction

Over thousands of years of human evolution and the advancement of knowledge, nature’s influence has spurred humans to synthesize novel and enhanced materials. Drawing inspiration from natural structures and architectures, these materials have propelled scientific progress into the realm of reticular chemistry [1,2,3]. In chemistry, ‘reticular’ describes the linking of building blocks via robust bonds, resulting in the creation of two-dimensional and three-dimensional networks [4]. Towards the end of the 20th century, the endeavors of scientists culminated in the development of a novel class of porous macromolecules composed of multiple building blocks [5,6,7,8]. Metal–organic frameworks (MOFs) were first synthesized by Yaghi et al. in the mid-1990s [9]. MOFs are formed through the self-assembly of central metal ions with organic ligands via coordination bonds [10]. MOFs possess outstanding properties like high porosity, superior electrical conductivity, catalytic activity, surface tunability, unsaturated metal sites, and mechanical flexibility, among other features [11]. Due to the high surface area and porosity of MOFs, they can be utilized as adsorbents [12]. However, MOFs exhibit low thermal stability and contain metal clusters and relatively unstable coordination bonds, which restrict their applications [13,14]. In response to this limitation, a new type of crystalline porous framework was synthesized [15].

COFs were initially synthesized in 2005 by Yaghi et al. [16]. These COFs are linked to light elements, such as H, B, C, O, and N, through strong bonds [17]. COFs possess remarkable properties, such as high thermal stability, high crystallinity, low density, and flexibility [18,19,20,21]. They were introduced as permanent porous frameworks in two forms: two-dimensional forms (2D) characterized by conjugated two-dimensional planes and three-dimensional forms (3D) maintaining the network structure through covalent bonds [22,23,24]. The 3D COFs are more effective than 2D COFs for material adsorption and removal due to their significantly larger internal surface area [25]. Among the various applications of COFs, gas separation [26], color removal [27], drug delivery [28], heterogeneous catalysts [29], conductivity [30], solar cells [31], electrochemistry [32], photoelectric [33], etc., are often utilized.

**Figure 1:**
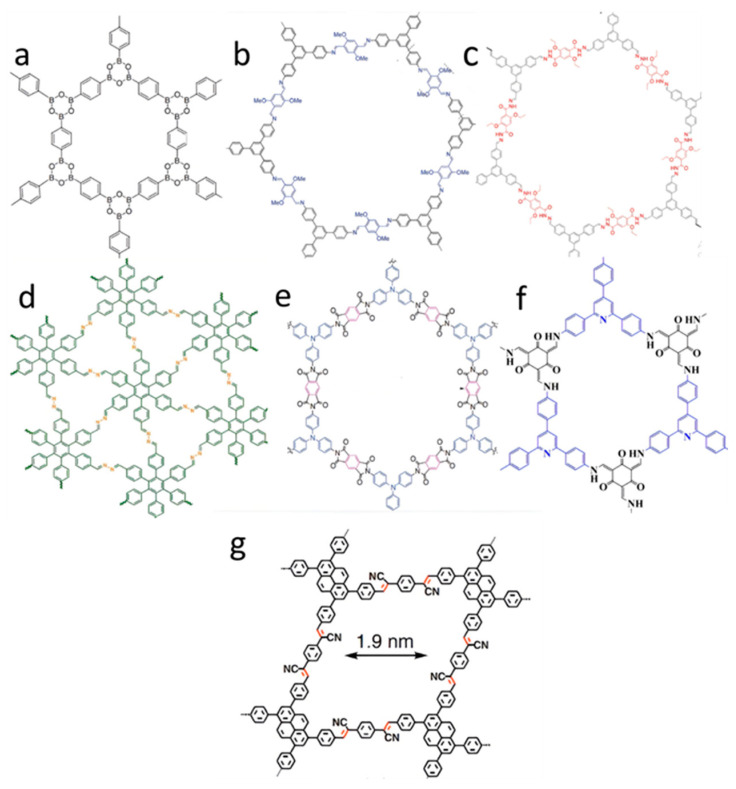
(**a**): Boron-based COFs [16], (**b**): imine-based COFs [34], (**c**): hydrazone-based COFs [35], (**d**): azine-based COFs [36], (**e**): imide-based COFs [37], (**f**): ketoenamine-based COFs [38], and (**g**): sp^2^ carbon-conjugated COFs [39].

COFs can be synthesized based on a variety of materials, some of which are as follows: boron-based COFs [16] (Figure 1a), imine-based COFs [34] (Figure 1b), hydrazone-based COFs [35] (Figure 1c), azine-based COFs [36] (Figure 1d), imide-based COFs [37] (Figure 1e), ketoenamine-based COFs [38] (Figure 1f), and sp^2^ carbon-conjugated COFs [39] (Figure 1g). COFs are more stable than their MOF counterparts; however, due to their higher hydrophobicity, they are less ideal in aqueous environments, which limits their applications in such settings. Therefore, researchers have used metallic nanoparticles on COF substrates to synthesize COF-containing metals, thereby enhancing the efficiency of COFs in various fields; one such field is catalysis, which the presence of metals particularly increased the efficiency of COFs in [40].

In today’s era, environmental pollution has become a significant challenge worldwide [41]. These pollutants can be categorized into various groups, including heavy metals and organic pollutants, among others [42,43]. Pollutants have been known to have adverse effects on the environment and human health, necessitating their removal for the well-being of both [44]. This need has prompted the establishment of research fields focusing on COFs as carbon supports for heterogeneous catalysts [45]. COFs are promising materials for addressing environmental issues due to their unique properties and structure [46].

The utilization of heterogeneous catalytic systems based on nanoparticles is highly efficient due to their surface-to-volume ratio and heightened chemical potential. Minimizing the aggregation of nanocatalysts is crucial for ensuring optimal catalyst performance, which has led to the utilization of COFs with a lasting porous structure as supports [47,48,49,50,51].

In line with these considerations, the objective of this research is to examine the reduction in nitrophenol, a pivotal organic pollutant, by employing COFs as a support material for noble metal nanoparticles.

## 2. Nitrophenols

Nitrophenols are rare compounds in nature that consist of a benzene ring, hydroxy, and nitro functional groups, with additional functional groups present in some of their derivatives [52,53]. These compounds exist in three isomeric forms: 2-nitrophenol (ortho-nitrophenol), 3-nitrophenol (meta-nitrophenol), and 4-nitrophenol (para-nitrophenol) [54]. Nitrophenols find applications in the synthesis of dyes (such as those used for leather, nylon, silk, and fur), fungicides, larvicides, herbicides, medicines, wood preservatives, and more [55,56,57]. They are present in groundwater, rainwater, clouds, fog, snow, car emissions, agricultural soil, and other environmental sources [58,59,60,61]. Additionally, they are compounds known for their high persistence, bioaccumulation, toxicity, carcinogenicity, hepatotoxicity, and genotoxicity, all of which the United States Environmental Protection Agency (USEPA) have classified as priority pollutants [62,63,64,65]. Nitrophenols can enter the human body through the inhalation of polluted air, consumption of contaminated water, and contact with dry soil surfaces [66].

To remove nitrophenol compounds, some traditional methods, such as ozonation and coagulation, have been reported, which require time and can be expensive [67]. Recently, with the help of COFs containing noble metal nanoparticles and a reducing agent, such as NaBH_4_, it has been found that nitrophenols can be reduced to aminophenols, leading to a lower degree of toxicity [68,69,70]. Aminophenols are used as synthetic intermediates in photographic industries, drug synthesis, electrochemical industries, and the preparation of dyes, among other applications [71,72,73,74].

## 3. Synthesis Techniques of COFs

Achieving highly ordered, stable, and crystalline COFs necessitates a deep understanding of the building blocks, synthesis techniques, and the thermodynamic principles that guide covalent bonding. Proficiency in these areas allows for the careful adjustment of synthesis conditions, which is vital for optimizing the structural and functional attributes of COFs. Numerous synthesis methods have been developed, each presenting unique advantages and particular challenges. Key techniques include solvothermal, mechanochemical, ionothermal, microwave, and sonochemical synthesis. By exploring these different strategies, researchers can tailor COF properties for specific applications, optimizing aspects such as pore size, surface area, and thermal stability.

### 3.1. Solvothermal Method

The usual method of solvothermal synthesis entails heating a mixture of monomers dissolved in a thoughtfully chosen blend of solvents within a sealed Pyrex tube at temperatures ranging from 80 to 120 °C for a period of 3 to 7 days. The solvent selection is critical as it enhances the solubility of the monomers. A combination of solvents can mediate the diffusion of monomers into the solution, which accelerates COF nucleation. Moreover, maintaining a sealed environment ensures that water is present during the reaction, promoting the effective crystalline growth of the COFs. This method has both advantages and disadvantages, with advantages such as the production of products with high crystallinity and the use of a wide range of solvents and disadvantages that include a long reaction time and the lack of use of insoluble monomers [75,76].

### 3.2. Mechanochemical Method

The mechanochemical method, employing a pestle and mortar for manual grinding, is receiving considerable attention for its simplicity, speed, and eco-friendliness, as it is solvent-free and suitable for room temperature operations. For example, COFs can be synthesized through Schiff base condensations at room temperature using this manual grinding technique. This method has both advantages and disadvantages. On the one hand, its environmental compatibility and the simplicity of the equipment used are notable benefits. On the other hand, it may face challenges related to uniformity and reproducibility [76,77].

### 3.3. Ionothermal Method

The ionothermal method employs molten salt or ionic liquid as a solvent and catalyst to generate solids at high temperatures and pressures. One of the main advantages of this technique is its ability to produce highly crystalline materials. However, the strict conditions required for this method can make it less popular, as this can limit the range of building blocks that can be used and may also result in unwanted degradation and side reactions, which can limit its applications [76,78].

### 3.4. Microwave Method

Microwave irradiation has gained significant attention as an alternative technique for COF synthesis. It presents multiple advantages compared to the solvothermal method, such as faster reaction times, purer products, and the ability to oversee phase behavior while concurrently managing temperature and pressure. However, it does have its limitations; it is not as easy to implement as other methods, is confined to specific COF types, and can face issues with thermal uniformity [79].

### 3.5. Sonochemical Method

Sonochemical synthesis is gaining traction as a preferred alternative to standard methods because of its rapidity and cost-effectiveness. This process involves ultrasound-induced cavitation, where bubbles grow and collapse in the solvent, generating extremely high local pressure and temperatures that accelerate chemical reactions [80].

## 4. Reducing Nitrophenols Using COFs Containing Noble Metal Nanoparticles

### 4.1. COFs Containing Gold (Au) Nanoparticle

Au nanoparticles have a significant ability to reduce 4-nitrophenol. The size of gold particles in the nanoscale, quantum effects, and structural effects can increase the activity of the catalyst. However, due to their high surface energy, gold nanoparticles easily accumulate, leading to a decrease in their catalytic activity. Therefore, COFs with high porosity, structural order, and easy access to their active sites can be used as a more efficient support compared to previous supports such as MOFs [81,82,83,84].

In 2014, Pachfule and coworkers discussed the first COF-supported Au nanoparticle catalyst [85]. This heterogeneous catalyst was named Au@TpPa and was used to reduce 4-nitrophenol to 4-aminophenol. The leaching test was used to prove the heterogeneity of the catalyst. The strong interaction between the support and the nanoparticles proved that the catalyst was heterogeneous. The good catalytic activity was due to the presence of COF as a support. High stability in organic, aqueous, acidic, and alkaline solvents could be mentioned as significant features of this COF.

In another study, a 2D TAPB-DMTP@Au catalyst was created (fabricated) [86]. Here, Au nanoparticles were modified using polyvinyl-pyrrolidone (PVP) and then encapsulated by COF. The presence of PVP enhanced the interaction between Au nanoparticles and COF to increase the reduction efficiency of 4-nitrophenol. According to the TEM images, after using the catalyst six times, the structure and order of the catalyst were still maintained, indicating the chemical stability of this compound and its recyclability.

Xu et al. [87] produced (fabricated) Fe_3_O_4_@TAPB-DMTP by grafting TAPB and DMTP monomers onto Fe_3_O_4_ nanoparticles. Finally, the Fe_3_O_4_@TAPB-DMTP-Au catalyst was created by adding gold nanoparticles. The structure of Fe_3_O_4_@TAPB-DMTP is core–shell, where Au nanoparticles are stabilized by an in situ reduction in HAuCl_4_ in the channels of the synthetic COF shell. The thermal stability of Fe_3_O_4_@TAPB-DMTP-Au as a catalyst was investigated at 200 °C for 4 h, and according to the results of FT-IR and nitrogen absorption, no noticeable changes in the structure of Fe_3_O_4_@TAPB-DMTP-Au were observed.

In another example, a propenyl-functionalized COF (A-COF) was first prepared using two structural units, 1,3,5-triformylbenzene and diethyl 2,5-bis(allyloxy)terephthalohydr-azide. Then, thiol groups were grafted onto the pre-synthesized A-COF by applying 1,2-ethanedithiol through a thiol-ene “click” reaction, resulting in the product called S-COF. By introducing Au nanoparticles to S-COF, the Au-S-COF target catalyst was synthesized for reducing 4-nitrophenol. The strong anchoring ability between Au nanoparticles and sulfur-containing groups grafted on the COF support facilitates the good dispersion and non-aggregation of Au nanoparticles, thereby enhancing the catalytic activity of the catalyst. Au-S-COF exhibited notable stability, as even after 10 cycles of use, its structure remained intact. Moreover, it demonstrated excellent recyclability, with its catalytic efficiency exceeding 90% after 10 uses. These two attributes are crucial for the practical application of catalysts [88].

**Figure 2:**
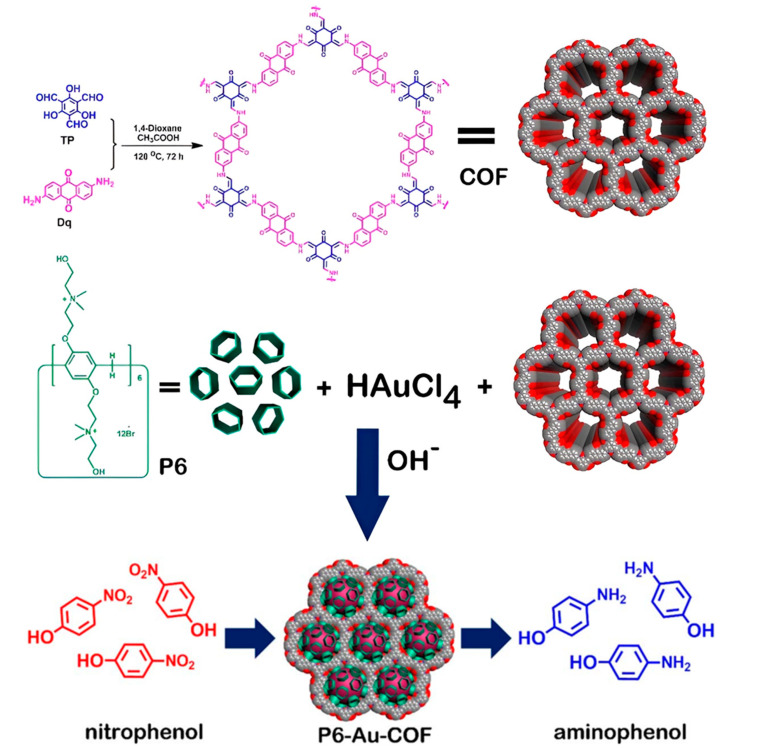
Synthesis of COF, P6-Au-COF nanomaterial, and its application in catalyzing a reduction in nitrophenol isomers [90].

Tan and co-workers synthesized P6-Au-COF [81] based on Dq and Tp as monomers of COF [89] and pillar [6] arene (P6) [90], which reduced Au nanoparticles (P6-Au) as a heterogeneous 2D nanocatalyst for reducing 4-nitrophenol to 4-aminophenol. The P6-Au-COF catalyst was prepared using a green and simple method. According to the Brunauer–Emmett–Teller (BET) method, the surface area of this composite decreased compared to the pure state of the synthetic COF. This decrease in surface area and pores indicated the anchoring of Au nanoparticles on the surface of the COF. In this composite, P6 served as a multifunctional macrocyclic supramolecular host capable of recognizing 4-nitrophenol, increasing its concentration around the catalyst, and accelerating the rate of the reaction. The conversion of 4-nitrophenol to 4-aminophenol was evident in the ultraviolet–visible (UV-Vis) spectroscopy adsorption spectrum. The decrease in the 400 nm peak corresponding to 4-nitrophenol and the increase in the 300 nm peak corresponding to 4-aminophenol were observed, indicating the catalytic function of (P6-Au-COF) in the reduction of 4-nitrophenol. Figure 2 illustrates the synthesis and application of (P6-Au-COF).

A functional COF was developed and used as catalytic support for reducing 4-nitrophenol to 4-aminophenol. For the synthesis of the desired catalyst, the pre-synthesized COF was immersed in methanol containing HAuCl_4_ to synthesize the Au@TpMA catalyst. According to the XRD results, the well-defined crystal structure of Au@TpMA was instrumental in improving catalytic activity, while the porous structure of COF prevented aggregation and a reduction in catalytic activity by facilitating the connection of Au nanoparticles. It is important to note that the catalytic property increased with the increase in Au nanoparticles up to a certain value. After reaching the maximum value, the catalytic property began to decrease due to the accumulation of Au nanoparticles. The optimal loading of Au nanoparticles on the COF substrate was found to be 40 mg. To understand the catalytic mechanism of Au@TpMA, comparative experiments were conducted without a catalyst, with the TpMA catalyst, and with the Au@TpMA catalyst. Only the Au@TpMA catalyst containing Au nanoparticles successfully reduced 4-nitrophenol to 4-aminophenol, causing the solution’s color to change from yellow to colorless. A positive aspect of this COF is its synthesis via the solid–solid method using a ball mill, which requires minimal solvent and time [91].

In this instance, two thiol-decorated COFs named SH-COF-1 [92] (the COF of which is composed of two monomers, DABDT and TBA) and SH-COF-2 (COF of which is composed of two monomers, DABDT and TFPB) were synthesized using the Schiff base condensation reaction with the solvothermal method. Based on the powder X-ray diffraction (PXRD) results, both COFs exhibit a layered and polycrystalline structure. The synthesized COFs could serve as a support to enhance the efficiency of Au nanoparticles as catalysts in the reduction of 4-nitrophenol to 4-aminophenol in water-using NaBH_4_. The rate of 4-nitrophenol reduction to 4-aminophenol was assessed via UV-VIS, where the intensity was directly proportional to the concentration of 4-nitrophenol. The catalyst’s recyclability after ten catalytic cycles exceeded 95%, indicating the excellent stability of this synthetic composite as a catalyst.

**Figure 3:**
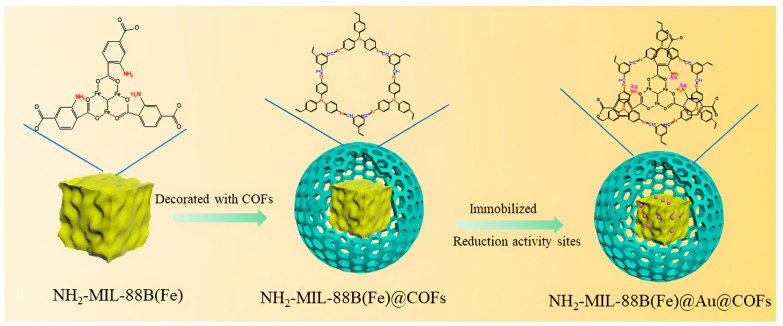
The fabrication of NH_2_-MIL-88B(Fe)@Au@COFs [93].

In 2023, Liu and his co-workers developed a MOF@Au@COF catalyst for reducing 4-nitrophenol [93]. They synthesized NH_2_-MIL-88B (Fe)@Au@COF for the first time, which utilized NH_2_-MIL-88B (Fe) as the core and Au@COF as the synthesized composite shell. In the reduction process, BH_4_^−^ was used as an electron donor and 4-nitrophenol as an electron acceptor, while Au nanoparticles accelerated the activity and overcame the kinetic energy barrier. According to (Figure 3), the COF was first synthesized, and then Au nanoparticles were added to it. As indicated by the transmission electron microscope (TEM) images, the good dispersion of Au nanoparticles on the COF support was the main reason for the catalytic activity of this compound to reduce 4-nitrophenol to 4-aminophenol. The good dispersion of Au nanoparticles, core–shell structure, excellent recycling activity, and exceptional thermal and chemical stability could be considered positive features of this catalyst.

### 4.2. COFs Containing Silver (Ag) Nanoparticles

Ag nanoparticles have been deposited onto porous substrates because of their stability, compatibility with the environment, and selectivity as a catalyst. The formation of hydrogen bonds between the substrate and Ag nanoparticles has led to better stabilization of the Ag nanoparticles [94,95,96].

Ag@TPHH-COF was studied by Wang and colleagues [97]. This catalyst was able to reduce nitroaromatic compounds, such as 4-nitrophenol. COF contains nitrogen and oxygen atoms that help anchor Ag nanoparticles more securely onto it, thereby enhancing the catalytic activity of these Ag nanoparticles. TPHH-COFs form a macromolecular structure through π-π interactions in a 3D structure. This catalyst exhibits several advantages, including the ability to reduce 4-nitrophenol and nitroaromatic compounds, as well as the rapid degradation of water pollutants.

In 2021, an Ag@SCOF catalyst was synthesized by attaching Ag nanoparticles to spherical COFs (SCOFs) [98]. The presence of nitrogen groups on COFs, made from two monomers, TPB and DVA, facilitated the adhesion of Ag nanoparticles to them. SCOF was performed as a semiconductor and co-catalyst, enhancing electron transfer and increasing the conversion rate of 4-nitrophenol to 4-aminophenol. TEM and XRD results confirmed the stability of the catalyst produced after a 24-h test, showing no degradation in its structure. This catalyst exhibited a suitable speed for catalytic regeneration; while its recovery capability is good, the process is complicated and costly, presenting a drawback for this catalyst.

**Figure 4:**
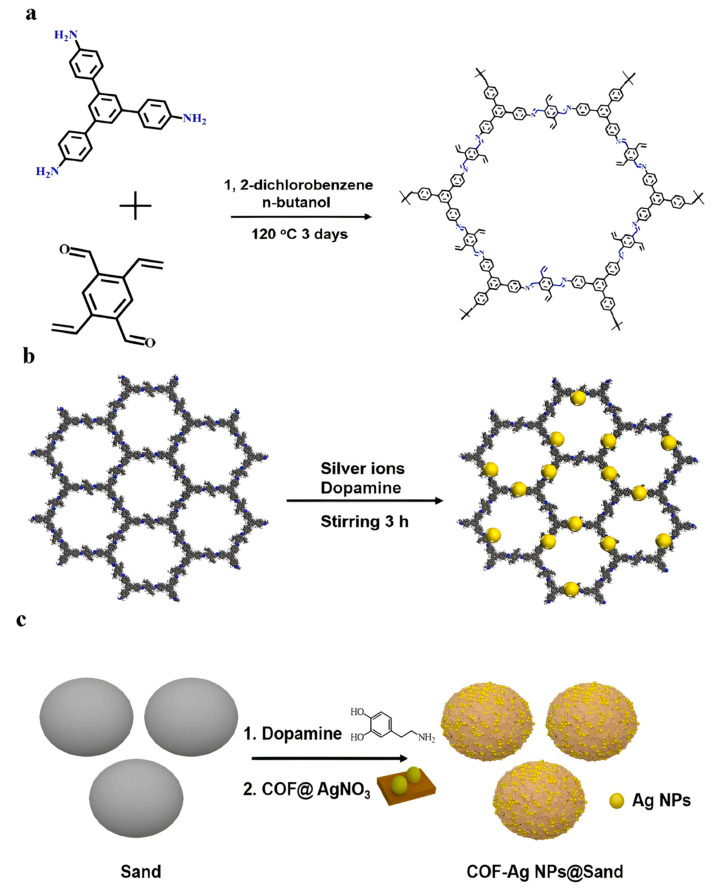
(**a**) Synthetic scheme of COF through the condensation reaction at high temperatures, (**b**) the fabrication of COF-AgNP nanocomposites, (**c**) the construction of the COF-AgNPs@Sand catalyst, including the first adhesion of sand and COF through PDA chemistry and then in situ reduction in silver ions and supporting AgNPs [100].

In this case, COF-AgNPs@sand used sand in addition to COF, which had a positive effect on the reduction in 4-nitrophenol due to its structure and properties. The sand used was modified with polydopamine and supported by Ag nanoparticles (Figure 4). This catalyst exhibited good catalytic activity for three reasons: (i) COF acted as a co-catalyst, facilitating electron transfer and providing active sites for the catalyst. (ii) Due to its structure and hydrophilic properties, sand allowed for the quick penetration of solutions. (iii) The fixation of COF-AgNP on sand with polydopamine increased the reduction efficiency to over 99% [99].

In another example, AgNPs@NCOFs were created [100]. The TAPA-PDA COF [101] creates numerous sites for nucleation because of the nitrogen in its structure. The available nitrogen forms connections between the Ag nanoparticles and the COF skeleton through interfacial interactions, causing a color change from red to yellow. This color change is the simplest way to observe the successful synthesis of AgNPs@NCOF. When COF is not used as a support, AgNPs tend to aggregate due to their high surface energy, leading to deactivation and a significant reduction in catalytic activity for 4-nitrophenol reduction. The rate constantly decreased by less than 6% after five catalytic cycles, indicating the excellent recyclability of this catalyst.

In 2022, Liu and colleagues separately synthesized two catalysts using Ag nanoparticles and Au nanoparticles alongside a suitable COF substrate [102]. These catalysts were named Au@C-PCTF and Ag@C-PCTF. PCTF was synthesized through the Friedel–Crafts reaction and utilized porphyrin as a building block [103]. Its porous structure facilitated the dispersion of metal nanoparticles as a substrate to enhance the catalytic activity of 4-nitrophenol reduction. Ag@C-PCTF exhibited higher catalytic activity than Au@C-PCTF since the substrate was the same for both catalysts. The enhanced activity of Ag@C-PCTF could be attributed to the smaller size of Ag nanoparticles and their better positioning on active sites. One of the factors contributing to the excellent stability of this catalyst could be the nitrogen atoms in the COF structure, which, through π-π interactions, facilitated the pre-concentration of 4-nitrophenol around the metal nanoparticles, thereby increasing the reaction rate.

Huang and colleagues synthesized AgNPs-3D-COF for the reduction in 4-nitrophenol and other applications [104]. The COF used as a support is 3D and contains hydroxyl groups, forming a diamond network structure (TFPM and DHBH are utilized as building units for this COF [105]. One key advantage of this COF is its three-dimensionality, which provides more surface area and active sites compared to 2D COFs. This feature is crucial for anchoring Ag nanoparticles, facilitating the accumulation of 4-nitrophenol on the COF, enhancing the connection between Ag nanoparticles and 4-nitrophenol, and promoting catalytic reduction. Oxygen atoms in the COF make it full of electrons, while 4-nitrophenol lacks electrons, causing them to interact naturally and convert 4-nitrophenol into 4-aminophenol. The effective coordination between Ag nanoparticles and the OH groups attached to the COF results in the high catalytic activity of AgNPs-3D-COF. Furthermore, the catalyst can be recycled due to its strong stability and the non-degradation of the COF.

This research outlines the development of a Schiff base covalent organic polymer (COP) synthesized through a condensation reaction involving 2,4,6-tris-(2-methoxy-4-formyl-phenoxy)-1,3,5-triazine and p-phenylenediamine. The resulting Ag-COP was employed as a catalyst for the reduction in nitroaromatics. The structure of the catalyst was confirmed using various analytical techniques. Findings show that Ag nanoparticles were uniformly distributed on the surface or embedded within the interlayer spaces of the COP. Ag-COP catalysts were identified as highly effective nanocatalysts for reducing nitroaromatics. The apparent rate kinetic constant (K_app_) and activity parameter (k’) were determined to be 0.0122 s^−1^ and 4.06 s^−1^ g^−1^, respectively. The catalyst achieved a 96.5% conversion rate after seven consecutive runs, maintaining its catalytic activity. This experimental study demonstrated the exceptional performance of COP nanoparticles as efficient materials for the removal of industrial contaminants [106].

### 4.3. COFs Containing Copper (Cu) Nanoparticles

Cu nanoparticles are a favorable option for 4-nitrophenol reduction reactions because of their electrical behavior, antibacterial properties, good stability, and cost-effectiveness compared to Au, Ag, and Pd nanoparticles [107,108]. The selection of COFs as a substrate for Cu nanoparticles is based on their semi-conductivity, which aids in electron transfer and ensures the appropriate anchoring of nanoparticles [109, 110].

TpPa-NH_2_ was chosen as a substrate for Cu nanoparticles [111], leading to the development of Cu-COF as a catalyst for the reduction in 4-nitrophenol. The presence of lone pairs of electrons in oxygen and nitrogen atoms within the COF structure enables interactions with Cu nanoparticles, resulting in the successful synthesis of the desired catalyst. Additionally, Cu nanoparticles interact with the oxygen and nitrogen atoms in their vicinity, compacting the COF structure more than usual. A noteworthy feature of this catalyst is the utilization of Cu nanoparticles instead of precious metals like Au, Ag, and Pd, making it economically viable. Moreover, its thermal stability extends up to 300 °C, rendering it highly suitable for catalytic applications.

**Figure 5:**
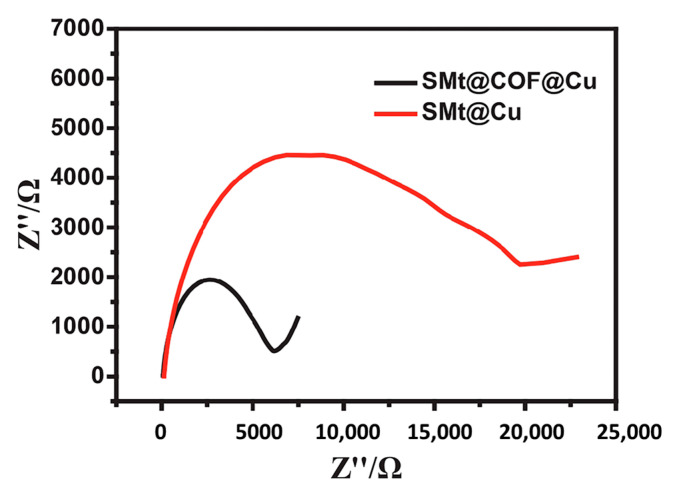
Electrochemical impedance spectroscopy of SMt@COF@Cu and SMt@Cu [112].

In another intriguing study, a synthetic COF was employed as a co-catalyst in the SMt@COF@Cu structure [112]. Initially, the COF was synthesized, followed by the doping of Cu nanoparticles onto it, and ultimately, spherical montmorillonite (SMt) was used as a support for COF@Cu. SMt@COF@Cu was utilized as a catalyst for reducing 4-nitrophenol. The self-sedimentation property of SMt facilitated easy separation from the mixture, allowing for its reuse, which is a significant and advantageous feature that addresses the catalyst recycling challenge. The COF within the structure enhances the reduction process and electron transfer, as illustrated in Figure 5 and confirmed by the comparison of electrochemical impedance spectroscopy between SMt@COF@Cu and SMt@Cu.

In 2020, CuFe_2_O_4_/Ag@COF was synthesized as a catalyst with a core–shell structure [113]. In this instance, the catalyst comprises COF (TAPB-DMTP) as a substrate and nanoparticles of Ag and magnetic CuFe_2_O_4_. The combined presence of Ag and CuFe_2_O_4_ enhances the catalytic activity. This catalyst exhibited structural order, high stability, and a suitable response to external magnetic fields, imparting positive characteristics. These include (i) an affinity for 4-nitrophenol through π-π interactions and its conversion to 4-aminophenol; (ii) thermal stability up to 300 °C; and (iii) rapid catalyst separation within a few seconds by applying an external magnetic field. Without COF as a substrate, the reduction process takes approximately 11 min, whereas, in the presence of COF, this time is reduced to 4 min, underscoring the significance of the COF in accelerating the reduction rate and preventing nanoparticle aggregation. COF also aids in pre-concentrating BH_4_^−^ and 4-nitrophenol, thereby expediting the reduction reaction. The synthesized catalyst shows promise for treating wastewater-containing nitrophenol.

COFs have been widely utilized as a support for incorporating nanoparticles and producing catalysts. For instance, Wu and colleagues synthesized Cu/Ag–COF through an in situ reduction and employed it as a catalyst for converting 4-nitrophenol to 4-aminophenol [114]. In their previous study, they utilized TPB and DVA monomers as COF building blocks [115]. In this setup, the coexistence of Ag and Cu nanoparticles enhanced the charge density, leading to a synergistic effect that significantly boosts both speed and catalytic activity. Analysis of the elemental composition confirms the presence of Ag and Cu nanoparticles on the spherical surface of COF, which validates the successful synthesis of this catalyst. The synthetically produced catalyst exhibited good thermal stability at temperatures below 400 °C. The conversion of 4-nitrophenol to 4-aminophenol using this catalyst offers three advantages: (i) enhanced catalytic activity stemming from the synergy of Ag and Cu nanoparticles, (ii) the prevention of nanoparticle aggregation facilitated by COF as a support, and (iii) an increased electron transfer rate attributed to the conductivity of COF.

**Figure 6:**
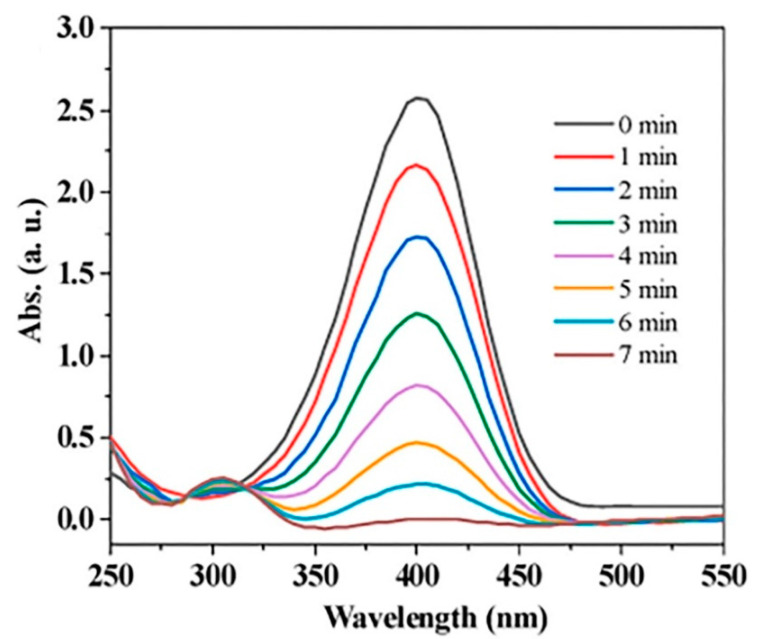
Time-dependent UV–vis spectra of the reduction in 4-NP catalyzed by Cu@TAPB-PCBA (0.4 mg/mL) [116].

In the latest study, Liu et al. synthesized Cu@TAPB-PCBA [116]. This catalyst swiftly reduced 4-nitrophenol to 4-aminophenol, a process monitored and confirmed through UV-Vis spectroscopy, which is displayed in Figure 6. The effective catalytic performance of this catalyst can be attributed to several factors: (i) Nitrogen atoms within the COF structure serve as nucleation sites for Cu nanoparticles. (ii) The remarkably small size of Cu nanoparticles facilitates efficient electron transfer, thereby promoting the reduction reaction of 4-nitrophenol. (iii) TAPB-PCBA features one-dimensional pores that restrict the aggregation of copper nanoparticles.

This investigation reports on the fabrication of a COP utilized as a support for Cu nanoparticle immobilization. Cu atoms were effectively chelated to the nitrogen and oxygen atoms present in the COP. The results indicate that Cu nanoparticles were uniformly and securely embedded into the COP’s surface. The Cu–COP composite showed significant catalytic activity in reducing 4-nitrophenol to 4-aminophenol. The calculated k’ and K_app_ for Cu–COP were 9.65 s^−1^ and 0.0193 s^−1^ g^−1^, respectively, with the complete conversion of 4-nitrophenol to 4-aminophenol achieved in just 3 min. Additionally, the nanoparticle conversion efficiency for the 4-NP reduction reached 97.1% over five cycles without any reduction in reactivity [117].

### 4.4. COFs Containing Palladium (Pd) Nanoparticles

Pd nanoparticles are commonly employed as catalysts in reduction reactions due to their high catalytic activity and excellent selectivity [118,119]. For instance, in a particular study, two catalysts—PtNPs@COF and PdNPs@COF—were synthesized [120]. These catalysts were produced using a similar method and exhibited the ability to convert 4-nitrophenol to 4-aminophenol. The COF structure incorporates the groups that serve to anchor and attach Pd or Pt nanoparticles to the COF substrate, thereby enhancing the catalytic activity of these catalysts. The notable advantage of the PdNPs@COF catalyst over the PtNPs@COF catalyst is its efficacy as a catalyst in the Suzuki coupling reaction, which is a crucial industrial process that has increased the utilization of PdNPs@COF.

In another study, a COF with a layered structure was utilized as a support for Pd nanoparticles. This composite was synthesized from TFB and PDA monomers using a one-pot method, resulting in the formation of Pd/COF-LZU1 [121]. The COF exhibited good order and crystallinity attributed to intramolecular hydrogen bonds and π-π aggregation. These characteristics facilitated the effective binding of Pd nanoparticles, subsequently enhancing the catalytic properties of Pd/COF-LZU1 in reducing 4-nitrophenol.

**Figure 7:**
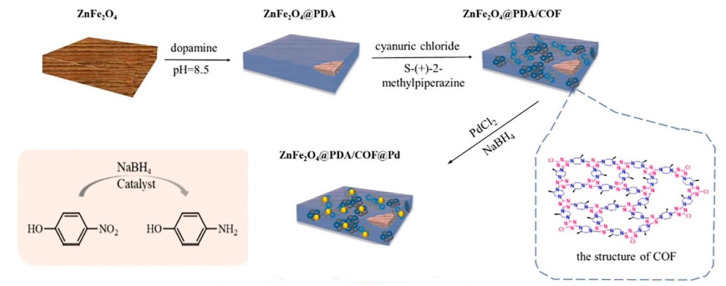
Schematic illustration of the synthesis and application of ZnFe_2_O_4_@PDA/COF@Pd [123].

ZnFe_2_O_4_@PDA/COF@Pd was synthesized by Lv et al. [122]. In this study, ZnFe_2_O_4_, characterized by a wood-like structure, was encapsulated in polydopamine, followed by the deposition of a COF layer as a platform for Pd nanoparticles. Subsequently, the introduction of Pd nanoparticles led to the development of a catalyst for the reduction in 4-nitrophenol. Notably, the size of the Pd nanoparticles exceeded the COF pores, resulting in their positioning between the layers of the COF. The abundance of nitrogen in the COF structure facilitated the strong adhesion of the Pd nanoparticles to the COF layers, thereby preventing their loss. Moreover, owing to its magnetic properties, the catalyst could be rapidly recovered using an external magnetic field, and even after five uses, its performance remained above 97%. Overall, the catalyst’s outstanding activity and stability can be attributed to (i) the presence of nitrogen atoms in the COF structure, enabling the effective binding of Pd nanoparticles, (ii) the interaction between Fe ions in ZnFe_2_O_4_ and polydopamine, and (iii) the placement of COF on polydopamine through a reaction involving chlorine atoms and amino groups (Figure 7) [123].

In 2022, COFa-2@Pd was synthesized and utilized as a catalyst for various applications [124], with a primary focus on reducing 4-nitrophenol to 4-aminophenol. The COF within the composite structure possesses a hollow spherical architecture, with its growth mechanism based on Oswald’s growth mechanism, along with allelic linkages that enhance its catalytic properties. The presence of an allelic structure promotes the orderly arrangement of Pd nanoparticles, leading to enhanced stability and excellent catalytic activity for this catalyst.

CS/ZIF-8@COFa-4@Pd was synthesized as a catalyst for reducing 4-nitrophenol [125]. According to scanning electron microscope (SEM) results, COFs were observed on the inner walls and channels of CS/ZIF-8. The successful synthesis of CS/ZIF-8@COFa-4@Pd was confirmed. In this structure, the presence of COF plays a crucial role as it prevents the leaching of Pd nanoparticles due to the nitrogen atoms in its structure. This maintenance of stability and activity of the catalyst helps prevent the swelling of CS/ZIF-8@Pd. After five cycles of the catalytic use of CS/ZIF-8@COFa-4@Pd, the loss of Pd nanoparticles was less than 0.08%, demonstrating the stability and excellent recyclability of this catalyst.

**Figure 8:**
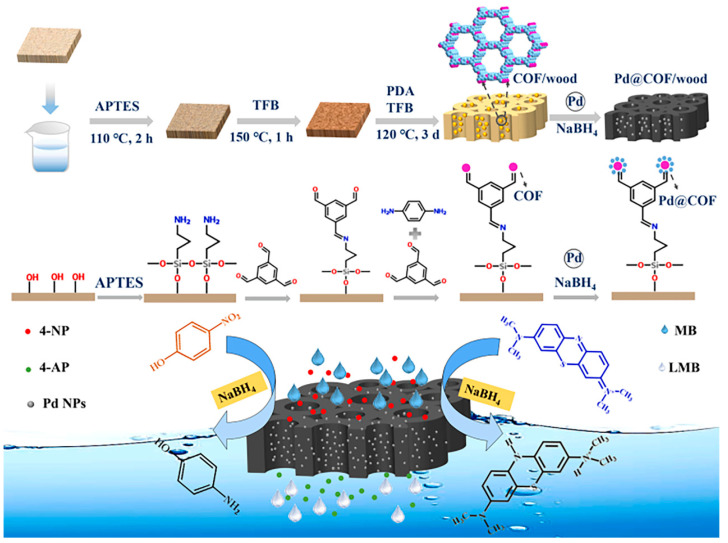
Schematic illustration of the preparation process of Pd@COF/wood and the catalytic reduction in 4-NP [126].

Pd@COF/wood, used as a catalyst, was synthesized by Cui et al. [126]. The COF integrated within the structure provides support for Pd nanoparticles, preventing their agglomeration. The utilization of porous wood aids in the uniform distribution of COFs, thereby enhancing their performance. The COF layers were synthesized on wood under solvothermal conditions, appearing yellow in color. By introducing Pd nanoparticles, Pd@COF/wood in black color was ultimately synthesized as a catalytic reactor (referred to Figure 8). The catalyst exhibited a notable reduction efficiency of 98.87% for converting 4-nitrophenol to 4-aminophenol within a mere 5 min. This high efficiency is considered a significant advantage of this catalyst.

MCM@Pd@COF, featuring a core–shell structure, was synthesized [127]. The COF, composed of two monomers, Tp and Tt, was arranged as a shell around the macroporous chitosan carbon microspheres and palladium (MCM@Pd). This setup serves to encapsulate and prevent the leaching of Pd nanoparticles, rendering them reusable. Thus, the encapsulation of MCM@Pd enhances the catalyst’s longevity. This composite is utilized as a catalyst with various applications, one of which is the reduction in 4-nitrophenol. Notably, MCM@Pd@COF exhibits a thermal stability below 150 °C, which is lower compared to the samples mentioned earlier, such as CuFe_2_O_4_/Ag@COF [113], Cu/Ag-COF [115], and SMt@COF@Cu [112]. This limitation hinders its use at high temperatures. Despite this, the catalyst has favorable applications in the Suzuki coupling reaction and in its effectiveness in reducing 4-nitrophenol.

## 5. Conclusions, Challenges and Future Perspectives

This review examines the reduction in nitrophenol by nanocatalysts using COF substrates. COFs enhance the catalytic activity of noble metal nanoparticles by serving as a suitable substrate due to their porous structure, active sites, and abundant functional groups. Within COFs, heteroatoms and functional groups establish a significant relationship with noble metal nanoparticles, strengthening their anchoring. These characteristics prevent the agglomeration of noble metal nanoparticles and enhance their catalytic activity. The use of these catalysts for removing nitrophenols as water pollutants offers a practical approach to purifying water contaminated with such compounds. Given the high reduction efficiency of these catalysts, COF-confined catalysts hold significant promise for the future, even though challenges persist regarding COFs. Similar to many advanced technologies, these nanocatalysts face various challenges that ongoing research should investigate. Firstly, modifying COFs after synthesis to enhance performance or introduce new functionalities can be difficult and may impact the overall structure of the compound. While COFs exhibit good stability, they can degrade over time in highly acidic or highly basic environments, and their prolonged use in such conditions faces significant challenges. Also, some COFs may undergo structural collapse or alteration during catalytic processes, affecting their functionality. One major issue is the lack of economical large-scale COF synthesis, which incurs high costs (for instance, the preparation cost of a COF sample amounts to about USD 3500/kg [128]). The presence of noble metal nanoparticles, such as gold, silver, and palladium, among others, has significantly increased synthesis costs. In the most recent study involving the viologen-derived COF-based metal-free catalyst [129], a catalyst devoid of base metal was employed, leading to substantial cost reductions and improved environmental friendliness. Additionally, the toxicity of released nanomaterials should be taken into account. Furthermore, the use of organic solvents in COF synthesis poses a significant challenge with adverse environmental effects. This issue could be mitigated by exploring alternative methods like solid–solid synthesis to minimize the use of such solvents [91]. Another crucial point is the efficiency of the reduction reaction of nitrophenols in real samples with complex matrices, making it challenging to definitively assess the catalysts’ reduction efficiency due to matrix effects. These challenges necessitate further research and development to enhance the practicality and effectiveness of COF-based catalysts in various applications.

The developmental horizon of COF nanocatalysts is bright. The continued evolution in COF design and synthesis may produce catalysts with improved activity and selectivity for specific reactions. Furthermore, current research emphasizes increasing the chemical and thermal stability of COFs, making them applicable in a wider range of industrial scenarios, even under extreme conditions. Additionally, advanced analytical approaches will provide better insights into COF structures at the molecular level, enabling more effective catalyst development. The integration of computational tools to predict COF properties and behaviors can also accelerate the discovery of new catalysts customized for particular applications, reducing the need for trial-and-error experiments. As research advances, COFs are expected to change the landscape of catalysis across various sectors, making significant contributions to the progress of chemistry and materials science. The proper selection of building blocks and the prediction of strong covalent bonds can lead to significant improvements in the chemical and thermal stability of COFs. Additionally, the presence of hydrophilic functional groups in the COF structural framework, along with metallic nanoparticles, enhances compatibility in aqueous environments. There is a bright future for various COF-based catalysts in wastewater treatment. For example, different types of COFs can be used to remove organic dyes, heavy metals, pesticides, pharmaceutical waste, and more. The use of COFs in wastewater treatment can also lead to reduced toxicity in treated effluents, which is important for environmental protection and compliance with regulations. Generally, COFs containing noble metal nanoparticles as catalysts demonstrate excellent thermal and chemical stability, high catalytic efficiency, and reusability. Despite the limited research on catalysts incorporating noble metal nanoparticles and COFs as substrates, it is believed that these catalysts play a significant role in reducing nitrophenols and purifying water sources.

## Data Availability

No new data were created or analyzed in this study. Data sharing is not applicable to this article.
